# Trophic interactions as determinants of the arbuscular mycorrhizal fungal community with cascading plant-promoting consequences

**DOI:** 10.1186/s40168-020-00918-6

**Published:** 2020-10-02

**Authors:** Yuji Jiang, Lu Luan, Kaijie Hu, Manqiang Liu, Ziyun Chen, Stefan Geisen, Xiaoyun Chen, Huixin Li, Qinsong Xu, Michael Bonkowski, Bo Sun

**Affiliations:** 1 State Key Laboratory of Soil and Sustainable Agriculture, Institute of Soil Science, Chinese Academy of Sciences, Nanjing, 210008 China; 2 College of Life Science, Nanjing Normal University, Nanjing, 210023 China; 3 College of Resources and Environmental Sciences, Nanjing Agricultural University, Nanjing, 210095 China; 4 Laboratory of Nematology, Wageningen University, 6700 ES Wageningen, Netherlands; 5 Institute of Zoology, Terrestrial Ecology, University of Cologne and Cluster of Excellence on Plant Sciences (CEPLAS), Cologne, Germany

**Keywords:** Arbuscular mycorrhizal fungi, Protists, Nematodes, AMF colonization, Phosphorus transporter genes, Plant productivity

## Abstract

**Background:**

The soil mycobiome is composed of a complex and diverse fungal community, which includes functionally diverse species ranging from plant pathogens to mutualists. Among the latter are arbuscular mycorrhizal fungi (AMF) that provide phosphorous (P) to plants. While plant hosts and abiotic parameters are known to structure AMF communities, it remains largely unknown how higher trophic level organisms, including protists and nematodes, affect AMF abundance and community composition.

**Results:**

Here, we explored the connections between AMF, fungivorous protists and nematodes that could partly reflect trophic interactions, and linked those to rhizosphere P dynamics and plant performance in a long-term manure application setting. Our results revealed that manure addition increased AMF biomass and the density of fungivorous nematodes, and tailored the community structures of AMF, fungivorous protists, and nematodes. We detected a higher abundance of AMF digested by the dominant fungivorous nematodes *Aphelenchoides* and *Aphelenchus* in high manure treatments compared to no manure and low manure treatments. Structural equation modeling combined with network analysis suggested that predation by fungivorous protists and nematodes stimulated AMF biomass and modified the AMF community composition. The mycorrhizal-fungivore interactions catalyzed AMF colonization and expression levels of the P transporter gene *ZMPht1;6* in maize roots, which resulted in enhanced plant productivity.

**Conclusions:**

Our study highlights the importance of predation as a key element in shaping the composition and enhancing the biomass of AMF, leading to increased plant performance. As such, we clarify novel biological mechanism of the complex interactions between AMF, fungivorous protists, and nematodes in driving P absorption and plant performance.

Video Abstract

## Introduction

The soil mycobiome contains functionally diverse fungi, many of which are notorious plant pathogens that reduce plant performance [1]. On the other end of the functional spectrum are mutualistic fungal taxa such as arbuscular mycorrhizal fungi (AMF). AMF are monophyletic in the phylum Glomeromycotina and form mutualistic associations with the vast majority of plant species including most economically important crops [2]. AMF enhance the plant nutrient availability, particular of phosphorus (P), due to the presence of a large interface for P acquisition via an extensive mycorrhizal mycelium [3]. Roots infected with AMF show an upregulation of high-affinity AMF-specific P transporter genes [4]. It is increasingly accepted that organic manure application shapes AMF community structure, and subsequently promotes AMF colonization, P absorption, and plant performance [5, 6]. However, this knowledge mostly stems from simplified controlled greenhouse experiments with little field-based evidence. The question remains whether these molecular mechanisms of the AMF community in mediating P absorption and plant productivity exist under organic farming systems.

Profound knowledge gains have been made on the fundamental processes that determine the structure of the mycobiome including AMF [7]. Abiotic parameters and plant species as bottom-up processes are crucial to structure AMF biomass and composition following organic fertilization. In contrast, top-down processes in structuring AMF have largely been ignored. Indeed, top-down predation by potentially fungivorous protists and nematodes are suggested to contribute to the turnover and changes in the structure and functioning of soil AMF community [8]. Importantly, most predators are not omnivorous but selective, which can influence fungal reproduction [9, 10]. Within trophic interactions, the direction and strength to which the predation of fungivores affect the AMF community remains a matter of debate. So far, scarce attention has been paid to the impact of trophic feeding on the AMF community, restricting our ability to better predict AMF dynamics in the rhizosphere.

As we are still limited in our knowledge of drivers of soil AMF, particularly the role of AMF-predators, we have an incomplete understanding of resulting functional consequences of these complex interactions. The distinct feeding preferences and selectiveness of fungivores on fungal diets can affect nutrient-dynamics and plant productivity [11]. It has been shown that predation by fungivorous nematodes on the AMF community changes P mineralization, ranging from negative to positive depending on host-identity and AMF growth rate [12]. A laboratory experiment revealed that protists increase plant performance and nutrient uptake by predation on bacteria and thereby enhancing AMF-regulated nutrient update [13]. Hitherto, the biological mechanism of predation-mediated nutrient acquisition of plants by fungivores in open-field environments is an open question in soil food-web research.

The intent of our study was to quantitatively assess the importance of predation on the AMF community and plant P uptake in comparison to the contribution of soil properties under field conditions. We performed a 17–year field experiment under four manure treatments in a low-fertile red soil (Acrisol). We asked the following three questions: (1) how do biomass, diversity and composition of the AMF community respond to manure treatments? (2) How and to what extent are fungivorous protists and nematodes linked to their potential prey AMF community? and (3) how do AMF-fungivore interactions mediate P uptake and plant productivity? Our work suggests that predation by fungivorous protists and nematodes positively regulates the biomass and composition of the AMF community, and subsequently promotes P uptake and plant productivity.

## Results

### Soil properties and phosphatase activities

One-way analysis of variance showed that manure treatments changed soil chemical properties (*P* < 0.01). High manure (M2 and M3) treatments were characterized by significantly (*P* < 0.001) higher soil pH, soil organic matter (SOM), total nitrogen (TN), and total phosphorus (TP) than the low manure (M1) and no manure (M0) treatments (Additional file 1: Figure S1). Similarly, available phosphorus (AP), nitrate nitrogen (NO_3_−N), and soil water content (SWC) were significantly (*P* < 0.01) elevated by high manure application. No significant differences of total potassium (TK, *P* = 0.317), available potassium (AK, *P* = 0.768), and ammonium nitrogen (NH_4_−N, *P* = 0.932) were detected between fertilization treatments. Alkaline phosphomonoesterase activity was increased with increasing levels of manure addition (*P* < 0.01). The M1 treatment possessed highest acid phosphomonoesterase activity with M2 and M3 treatments having lowest levels (Additional file 1: Figure S2).

### Plant growth, root morphology, and P transporter

**Fig. 1:**
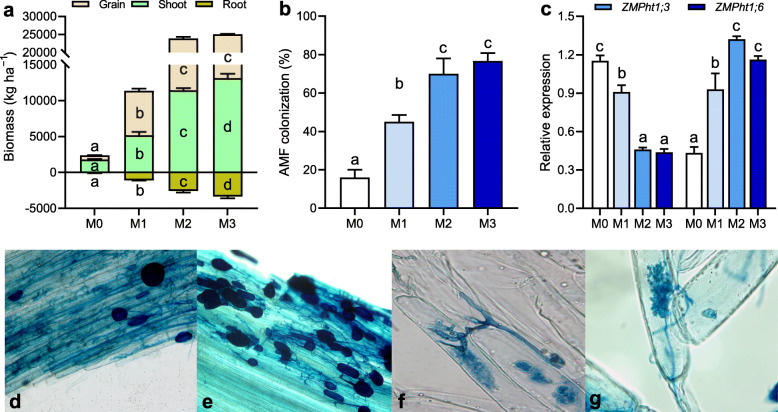
Plant growth, root morphology, and phosphorus transporter in the rhizosphere under manure treatments. **a** Plant productivity. **b** Root colonization frequencies by arbuscular mycorrhizal fungi (AMF). **c** The expression of P transporter genes (*ZmPht1;3* and *ZmPht1;6*) in the PHT1 family. Plant productivity is the sum of root, shoot, and grain biomasses. **d**, **e** AM fungal hyphae and vesicles at × 100 magnification in maize roots. **f**, **g** AM fungal arbuscules at × 400 magnification in maize roots. Bars (*n* = 3) with different lowercase letters indicate significant differences as revealed by Tukey’s HSD tests (*P* < 0.05). M0, no manure; M1, low manure; M2, high manure; M3, high manure plus lime

Root morphology was significantly (*P* < 0.001) affected by manure treatments, such that root dry biomass, root length, projected area, surface area, average diameter, root volume, tips, forks, and crossings exhibited a general trend of M3 ≥ M2 > M1 > M0 (Additional file 1: Table S1). The same trend was found for plant productivity, including shoot biomass, root biomass, and grain yield of maize (*P* < 0.001, Fig. 1). M2 and M3 treatments significantly increased root colonization frequencies by AMF compared to the M0 treatment (*P* < 0.001, Fig. 1). The expression of the P transporter gene *ZmPht1;6* was upregulated by 3.0 and 2.7 times under the M2 and M3 compared with the M0 treatment (*P* < 0.001, Fig. 1). However, the starvation-inducible P transporter (*ZMPht1;3*) of the PHT1 family showed an opposite expression pattern as it significantly decreased under high manure treatments (*P* < 0.01).

### Communities of AMF and saprotrophic fungi in the rhizosphere

**Fig. 2:**
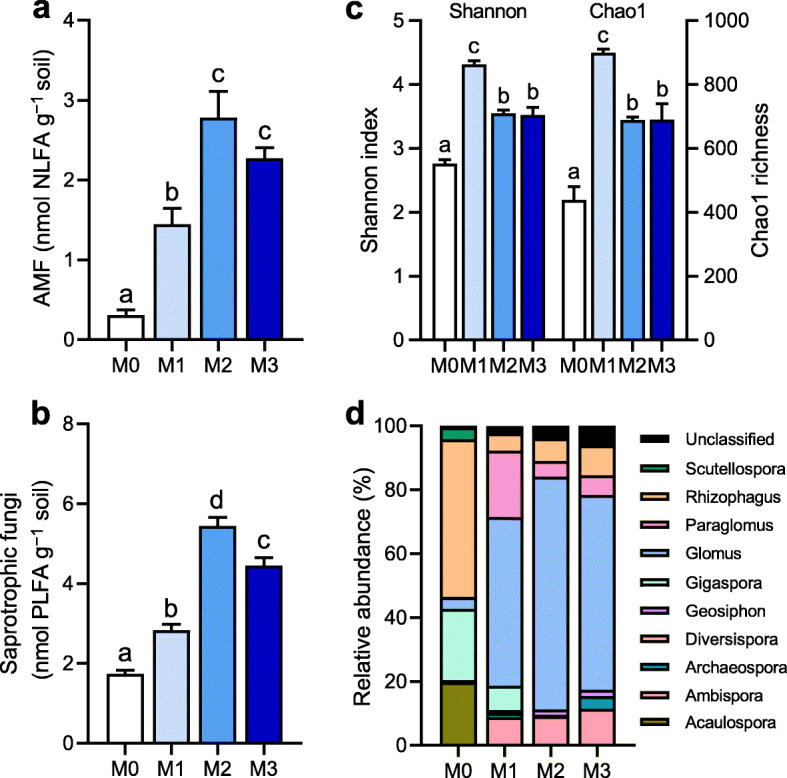
The communities of arbuscular mycorrhizal fungi (AMF) and saprotrophic fungi in the rhizosphere under manure treatments. **a** AMF biomass. **b** Saprotrophic fungal biomass. **c** Diversity. **d** AMF community composition. Bars (*n* = 3) with different lowercase letters indicate significant differences as revealed by Tukey’s HSD tests (*P* < 0.05). NLFA, neutral lipid fatty acid; PLFA, phospholipid fatty acid. M0, no manure; M1, low manure; M2, high manure; M3, high manure plus lime

We determined the biomasses of AMF and saprotrophic fungi in the rhizosphere by NLFA and PLFA analysis. On average, the biomass of saprotrophic fungi was 3.1 times higher than AMF biomass (*P <* 0.0001). The biomasses of AMF and saprotrophic fungi under the M2 and M3 treatments were 3.3−4.6 times and 2.6−3.1 times than those under the M0 treatment (Fig. 2, *P* < 0.05). Illumina sequencing to investigate AMF diversity indicated that the Shannon index and Chao1 richness was significantly higher under the M2 and M3 treatments than under the M0 treatment, but lower than under the M1 treatment (Fig. 2). Across all samples, the AMF community consisted of the dominant genera *Glomus* (47.6%), *Rhizophagus* (17.8%), *Paraglomus* (8.0%), *Gigaspora* (7.5%), *Ambispora* (7.4%), and *Acaulospora* (5.0%), followed by the rare genera *Archaeospora* (1.5%), *Scutellospora* (1.2%), *Geosiphon* (1.1%), and *Diversispora* (0.11%) (Fig. 2). The comparison of AMF community composition by principal coordinates analysis showed a significant (*P* < 0.01) separation among four manure treatments (Additional file 1: Figure S3). There were significantly larger abundances of *Ambispora*, *Glomus*, and *Paraglomus* under manure treatments in comparison with the M0 treatments, while *Acaulospora*, *Gigaspora*, and *Rhizophagus* displayed the opposite trends (*P* < 0.05). The ratio of AMF to plant biomass was significantly lower under the high manure treatments than under the M0 and M1 treatments (Additional file 1: Figure S4). PERMANOVA indicated that manure treatments explained approximately two-thirds (67.8%) of the variations in AMF community composition (*P* < 0.001). Biomass and composition of the AMF community were positively correlated with AMF colonization frequencies, alkaline phosphatase activity, and expression of *ZmPht1;6* gene, respectively (Additional file 1: Figure S5).

### Communities of fungivorous protists and nematodes in the rhizosphere

**Fig. 3:**
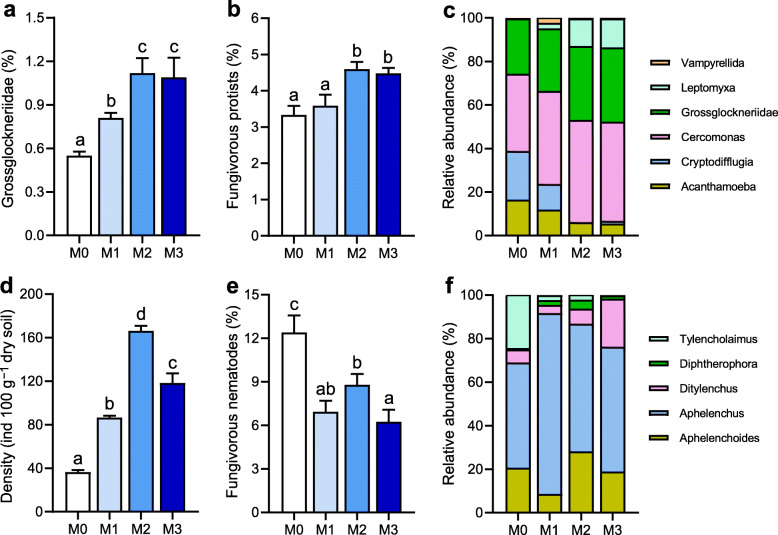
The assemblages of fungivorous protists and nematodes in the rhizosphere under manure treatments. The relative proportion of Grossglockneriidae to all protists (**a**) and fungivores to all protists (**b**), assemblage of fungivorous protists (**c**), the density of fungivorous nematodes (**d**), the relative proportion of fungivores to all nematodes (**e**), and assemblage of fungivorous nematodes (**f**). Bars (*n* = 3) with different lowercase letters indicate significant differences as revealed by Tukey’s HSD tests (*P* < 0.05). M0, no manure; M1, low manure; M2, high manure; M3, high manure plus lime

**Fig. 4:**
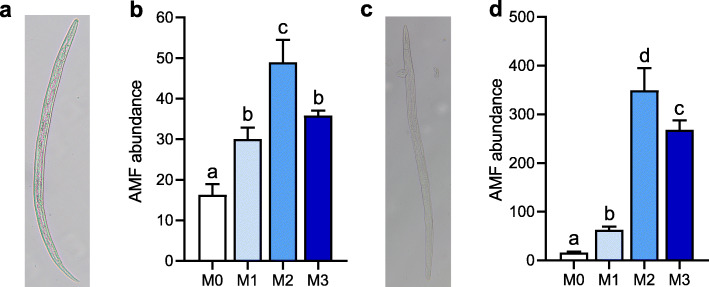
AMF abundance inside the body of the two dominant fungivorous nematodes *Aphelenchoides* (**a**, **b**) and *Aphelenchus* (**c**, **d**). **a** and **c**, × 200 magnification. AMF abundance inside *Aphelenchoides* and *Aphelenchus* was expressed as copy numbers of the AMF 18S rRNA gene per nematode, respectively. Bars (*n* = 3) with different lowercase letters indicate significant differences as revealed by Tukey’s HSD tests (*P* < 0.05). M0, no manure; M1, low manure; M2, high manure; M3, high manure plus lime

As for fungivorous protists in the rhizosphere, PCoA, and PERMANOVA indicated that the community compositions were determined by manure treatments (Additional file 1: Figure S3, 53.8%, *P* < 0.001). Overall, the obligate fungal-feeding family Grossglockneriidae (31.1%) was dominant among the identified fungivorous protists (Fig. 3). Dominant facultative fungivorous genera were *Cercomonas* (43.7%) and *Acanthamoeba* (10.3%), cumulatively representing 54% of the potentially fungivorous protists identified (Fig. 3). The relative proportion of Grossglockneriidae to all protists and fungivores to all protists under the high manure treatments significantly exceeded those under the M0 and M1 treatments (Fig. 3, *P* < 0.05). Similar to fungivorous protists, PERMANOVA combined with PCoA indicated that the assemblages of fungivorous nematodes varied by manure treatments (Additional file 1: Figure S3, 69.9%, *P* < 0.001). From the fungivorous nematodes, the genera *Aphelenchus* (61.9%) and *Aphelenchoides* (19.1%) were the two most abundant groups in the nematode assemblages (Fig. 3). The density of fungivorous nematodes under M2 and M3 treatments was 3.3−4.6-folds higher than that under the M0 treatment, mainly caused by the increase of the dominant genera *Aphelenchoides* (3.0−6.2-folds) and *Aphelenchus* (3.8−5.5-folds). However, the relative proportion of fungivorous nematodes to all nematodes and the ratio of fungivorous nematodes to plant biomass were substantially decreased under manure treatments compared to the M0 treatment (Fig. 3, Additional file 1: Figure S4). Notably, AMF abundance inside *Aphelenchoides* and *Aphelenchus* was significantly higher under the M2 (48.9 ± 5.6 and 349.1 ± 45.9 copies per nematode) and M3 (35.8 ± 1.3 and 268.2 ± 19.5 copies per nematode) treatments compared to the M0 (16.3 ± 2.6 and 15.9 ± 2.2 copies per nematode) and M1 (30.0 ± 2.9 and 62.5 ± 6.9 copies per nematode) treatments (Fig. 4). The fungivorous protists (*Cryptodifflugia*, Grossglockneriidae, and *Leptomyxa*) and nematodes (*Aphelenchoides* and *Aphelenchus*) were significantly associated with the biomass and composition of the AMF community, as well as the biomass of saprotrophic fungi (Additional file 1: Figure S5).

### Soil properties, AMF, and fungivorous protists and nematodes jointly mediated P availability and root P transporter gene

**Fig. 5:**
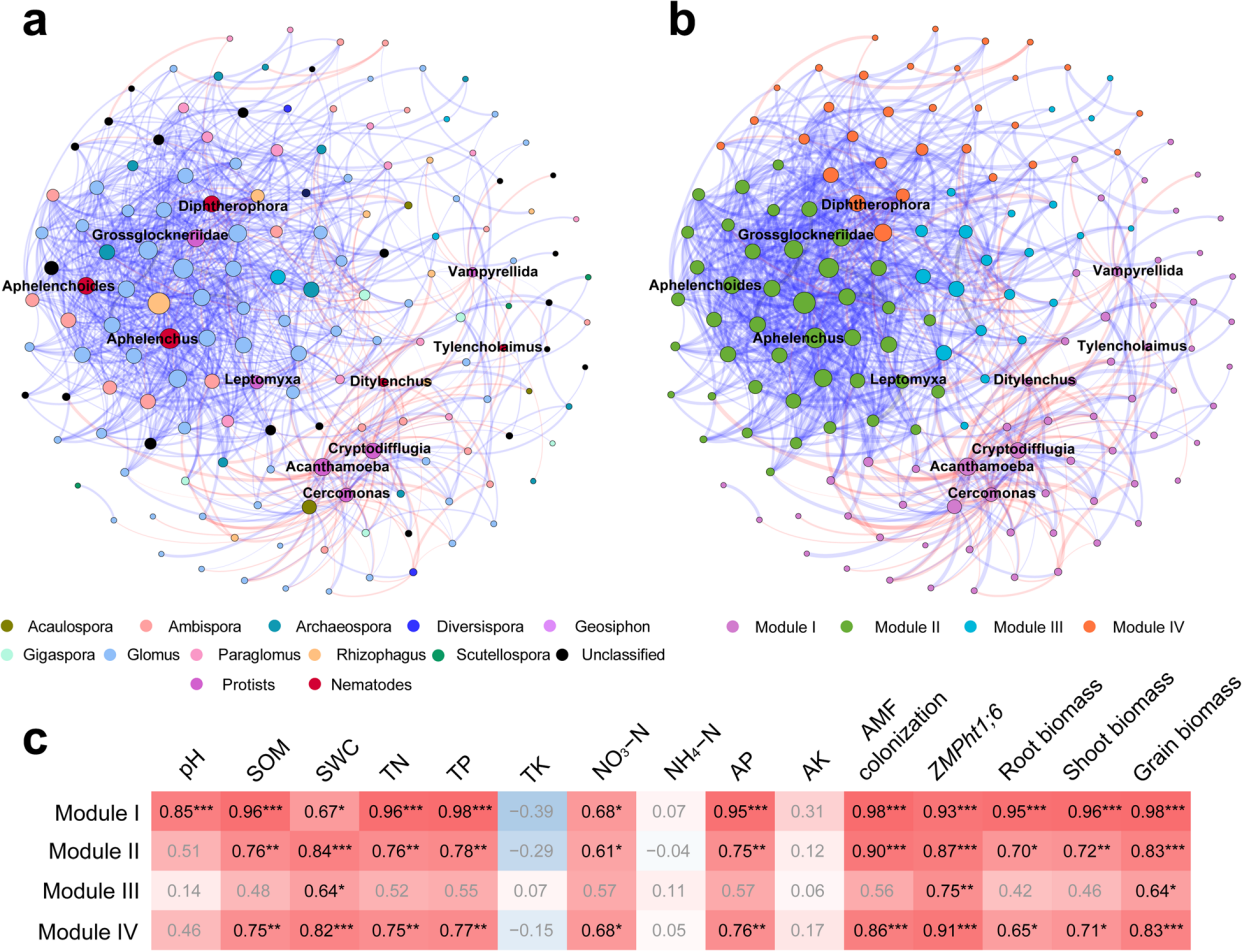
Co-occurrence network of rhizosphere soils showing strong and significant correlations. The network is colored by AMF/protist/nematode taxa (**a**) and modules (**b**) with all the fungivorous protists and nematodes being listed, respectively. Modules I−IV represent four clusters with closely interconnected nodes. Size of each node is proportional to the number of connections (degree), and the thickness of each connection between two nodes (edge) is proportional to the value of correlation coefficients. Blue edges indicate positive, red edges negative connections. **c,** Correlation coefficients between module eigengenes, soil properties, AMF colonization, expression of *ZMPht1;6* gene, and plant productivity. Bold values denote significant relationships. SOM, soil organic matter; SWC, soil water content; TN, total nitrogen; TP, total phosphorus; TK, total potassium; NH_4_−N, ammonia nitrogen; NO_3_−N, nitrate nitrogen; AP, available phosphorus; AK, available potassium. ***P < 0.001; **P < 0.01; *P < 0.05

AMF, mycophagous protists, and fungivorous nematodes were clustered into four distinct modules in co-occurrence networks, which we examined to decipher module-trait relationships (Fig. 5). Modules I, II, and IV consisted of 63, 42, and 31 nodes, involving AMF, fungivorous protists, and nematodes, respectively, whereas module III comprised 21 members exclusively from AMF. Modules II and IV displayed more positive correlations (411 and 69 edges) than negative correlations (0 and 5 edges). However, the ratios of negative correlations (73 edges) to positive correlations (92 edges) were increased in module I compared to modules II and IV. Modules I, II, and IV were positively correlated with soil chemical properties, including SOM, TN, TP, AP, SWC, and NO_3_−N (*r* = 0.61−0.98, *P* < 0.05). Moreover, these three modules were positively associated with alkaline phosphatase activity (*r* = 0.83−0.94, *P* < 0.001), AMF colonization frequencies (*r* = 0.86−0.98, *P* < 0.001), and the AM-specific P transporter gene *ZmPht1;6* expression (*r* = 0.87−0.93, *P* < 0.001), rather than acid phosphatase activity (*P* > 0.05) (Fig. 5).

**Fig. 6:**
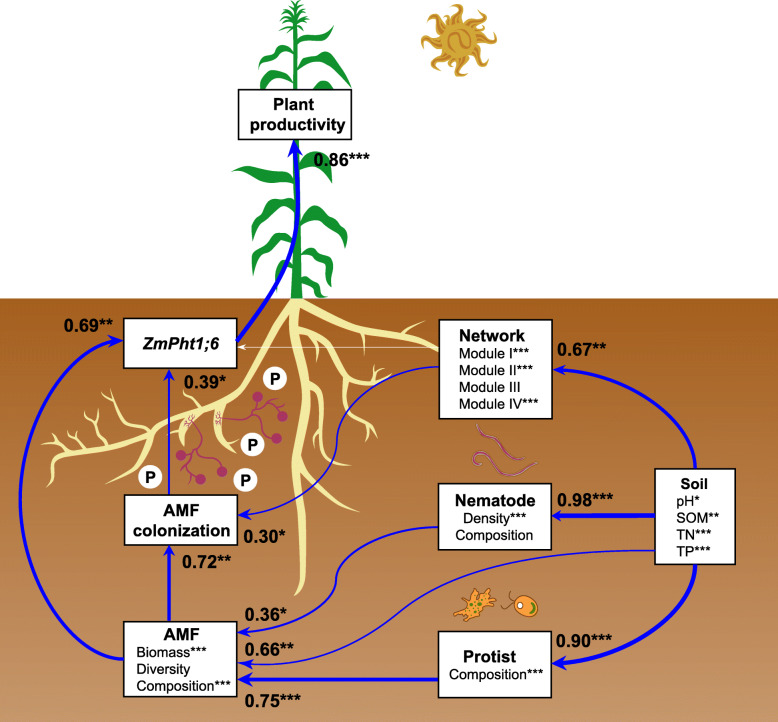
Impacts of soil variables, arbuscular mycorrhizal fungal (AMF) community, protists, and nematodes on AMF colonization, phosphorus transporter (expression of P transporter gene *ZMPht1;6*), and plant productivity as estimated using structural equation modeling (SEM) analysis. Based on random forest analyses, the significant predictors were chosen to perform the SEM analysis. The latent variables (soil properties, the AMF, protist and nematode community, and networks) inside the boxes were used to integrate the effects of multiple conceptually-related observed variables into a single-composite effect. Each observed variable is shown as normal font. Soil properties are represented by pH, soil organic matter (SOM), total nitrogen (TN), and total phosphorus (TP). The AMF community is represented by biomass, diversity (Shannon index), and composition (first principal coordinates, PCoA1). The protist community is represented by composition (PCoA1). The nematode community is represented by density and composition (PCoA1). The trophic co-occurrence networks between AMF, protist, and nematodes is represented by four modules indicated as module eigengenes. Plant productivity is the sum of root, shoot, and grain biomasses. Blue lines indicate positive relationships, and numbers associated with lines indicate the correlation coefficient. Widths of arrows indicate strength of significant standardized path coefficients. Paths with non-significant coefficients are presented as white lines. ***P < 0.001; **P < 0.01; *P < 0.05

Random forest modeling indicated that soil pH (*P* < 0.05), SOM (*P* < 0.05), TN (*P* < 0.01), and TP (*P* < 0.01) were the primary predictors among soil abiotic variables for AMF colonization and *ZmPht1;6* gene expression (Additional file 1: Figure S6). As for biotic variables, variations in AMF colonization and expression of *ZmPht1;6* gene were significantly affected by the biomass and composition of the AMF community (*P* < 0.01), and by the assemblages of fungivorous protists and nematodes (*P* < 0.05). Structural equation modeling (SEM) further predicted that AMF colonization and expression of *ZmPht1;6* gene were directly influenced by the AMF community and indirectly by the assemblage of fungivorous protists and nematodes (Fig. 6). Compared to the AMF community, mycorrhiza-fungivores networks had a significant, albeit weaker contribution to AMF colonization, and consequently showed a significantly indirect relationship with the expression of *ZmPht1;6* gene and plant productivity.

## Discussion

### Manure treatments shaped biomass, composition, and functioning of the AMF community

Overall, we showed that manure application increased the biomasses of AMF and saprotrophic fungi. Particularly, modifications of pH, SOM, TN, and TP under manure amendments may promote AMF root colonization by enhancing AMF spore germination and growth rates. Organic fertilization elevates AMF abundance and shapes the community, which may facilitate symbiose between AMF and plants [14]. The presence of mycorrhiza may favor plant nutrient acquisition by speeding up transformation of organic manure [15]. Particularly *Glomus* increased in manure treatments, a cosmopolitan, and abundant AMF genus in natural and agricultural soils [16, 17]. This pattern can be explained by the fact that *Glomus* is efficient in acquiring N and P [18, 19]. The observed overall AMF community changes can mediate P cycling rates and facilitate P availability for plants [20]. The upregulation of the AM-inducible P transporter (*ZMPht1;6*) implies a possible functional shift from direct to mycorrhizal-dependent P uptake under high manure application. AMF in symbiosis are reported to induce plant Pht1 transporters and switch to a mycorrhizal-dependent P uptake [21]. Accordingly, our results suggest that there is a positive linkage between soil nutrients, AMF, and plant colonization under increased manure application. These cascading effects of P acquisition can have a positive consequence on plant biomass and crop yield.

### Predation by fungivorous protists and nematodes mediated AMF community

Obligate and potentially facultative fungivorous protists and nematodes were present in all soils and were linked to fungal community shifts, at least partly by increasing AMF abundance and shaping AMF community composition (Fig. 6, Additional file 1: Figure S5). Protists and nematodes are suggested to be important top-down structuring elements of fungal communities [9, 10], a notion here extended to AMF specifically. We observed a significant decrease in the ratios of AMF to plant productivity and fungivores to plant productivity with increasing manure input, suggesting that the alteration in the AMF and fungivorous nematode communities was independent of plant effects with manure addition. AMF abundance inside two dominant fungivorous nematodes (*Aphelenchoides* and *Aphelenchus*) was significantly elevated in the high manure treatments (Fig. 4), providing empirical evidence for predation by nematodes on AMF. Indeed, the mycelium and spores of AMF and saprotrophic fungi can be preyed upon by fungivores nematodes [22] and likely protists [23]. Species-specific selective predation might influence the biomass and structure of the associated AMF community, as well as the biomass of saprotrophic fungi. For example, a specialized feeding structure allows the obligate fungivores Grossglockneriidae to perforate hyphae and thereby prey specifically on hyphal-forming fungi [24, 25]. The facultative fungivores including *Cercomonas* and *Leptomyxa* can consume yeast stages and spores of fungi [26]. Fungal-feeding nematode genera *Aphelenchoides* and *Aphelenchus* multiply with mycelium of *Glomus* [27], and likely facilitate the development of external hyphae [28, 29]. Populations of *Aphelenchus* sp. might induce a more strongly positive impact on AMF than *Aphelenchoides* due to higher feeding rate (Fig. 4). Close relationships between AMF, protists, and nematodes in co-occurrence networks imply complex connectedness through direct and indirect trophic interactions. Positive connections between AMF and fungivores suggest that the germination of spores and radial growth of rhizosphere AMF species increases under predation by fungivorous protists and nematodes [8]. The positive effects of predation on the development of AMF hyphae may be partly responsible for recycling minerals locked up in senescent fungal tissue and providing high lipid contents in spores [30, 31]. Another possible explanation is that the presence of fungivorous protists and nematodes created an additive positive effect on AMF biomass and activity, by preferentially feeding on non-active parts of the AMF community or/and on competing non-AMF species. However, we acknowledge that the linkage between AMF and fungivores is bidirectional rather than unidirectional. As fungivores have been shown to directly prey on fungi to maintain their survival, it indeed is fungi who determine the density of fungivores protists and nematodes.

### Interactions between AMF, fungivorous protists, and nematodes contributed to plant phosphorus absorption and productivity

We argue that fungivorous protists and nematodes modulate beneficial plant–AMF interactions by changing the functionality of the rhizosphere AMF community (Fig. 6). This contradicts the classical perceptions that protists and nematodes are less important in the fungal- than in the bacterial-energy channel [32]. However, several studies have supported that this might be a misconception as the biomass of fungivorous protists can equal that of bacterivorous protists [33], and many previously suggested bacterivorous protists are actually feeding also on fungi [34]. Taking this into consideration, we provide further evidence for the importance of predation by smaller organisms in the fungal-energy channel and expand it to the ecologically and economically plant-relevant fungal subgroup AMF. AMF induce the formation of large soil aggregates, which provides more suitable microhabitats for the survival and predation of fungivores under manure treatments [35, 36]. Tight correlations between AMF, protists and nematodes suggest that a complex community structure with many linkages is present in the soil food web [37]. Particularly, the association between AMF and fungivores in special functional modules in our network analysis might contribute to increasing AMF colonization and plant performance. Predators can affect prey abundance and activity via consumptive effects, suggesting that predators recycle immobilized nutrients to control prey functioning [11]. Furthermore, fungivorous protists and nematodes promoted soil alkaline phosphatase activity linked to mineralization of fresh organic manure, and recycled phosphorus dynamics to enhance host-nutrient availability by the predation on the AMF community. This was likely caused by trophic interactions, resulting in accelerated soil P solubilization processes, root P uptake, and plant productivity. However, we cannot fully evaluate the unidirectional link that top-down predation affects AMF communities as also bottom-up links likely contribute to the observed patterns. Potential mechanisms of the positive interplay between fungivores, AMF, and plants still require further mechanistic investigation taking account of the whole soil microbiome.

## Conclusions

Our study provides novel insights on the importance of links between fungivorous protists and nematodes with AMF that likely leads to cascading effects on plant P uptake and enhanced crop production. We conclude that fungivorous protists and nematodes likely play a key role in structuring the soil mycobiome in favor of a plant-beneficial AMF community. Future research should continue to take advantage of additional approaches such as stable isotope tracking to experimentally determine AMF-fungivore linkages. If confirmed, our findings hold great promise in targeted microbiome manipulation for increased crop production through the application of fungivorous protists and nematodes.

## Methods

### Field experiment description

The long-term fertilization experiment commenced at the Yingtan National Agroecosystem Field Experiment Station of the Chinese Academy of Sciences (28°15′20′′ N, 116°55′30′′ E) in Jiangxi Province, China. The experiment site has a typical subtropical climate with a mean annual temperature of 17.6 °C and precipitation of 1795 mm. The soil is classified as Ferric Acrisol according to the Food and Agricultural Organization (FAO) classification system. The long-term field experiment followed a completely randomized design with three replicates. The experiment was conducted since 2002, which consisted of 12 concrete plots with the following size: 2 m long, 2 m wide, and 1.5 m deep. The four manure treatments were (1) no manure (M0); (2) low manure with 150 kg N ha^−1^ years^−1^ (M1); (3) high manure with 600 kg N ha^−1^ years^−1^ (M2); and (4) high manure with 600 kg N ha^−1^ years^−1^ and lime applied at 3000 kg Ca (OH)_2_ ha^−1^ 3 years^−1^ (M3). Pig manure had an average total carbon content of 386.5 g kg^−1^ and a total nitrogen content of 32.2 g kg^−1^ on a dry matter basis. The field was planted annually with corn monoculture (cultivar Suyu No. 24) from April to July. Each plot was grown with 20 maize plants. No management measures were taken with the exception of weeding by hand.

### Soil sampling, physicochemical properties and phosphomonoesterase activity assays

Soil samples from each plot were collected after the harvest in late July 2018. In each plot, 10 soil cores were collected from the surface layer (0−20 cm) using a Dutch auger (5 cm diameter), and were mixed to form a composite sample. After field collection, fresh samples were placed on ice and immediately transported to the laboratory, where they were sieved (2 mm) to remove visible residues and then homogenized. Soil samples were subdivided into three subsamples for analyzing soil physicochemical properties, the AMF community, the assemblages of fungivorous protists, and nematodes.

Soil pH was measured by a glass electrode with water:soil ratio of 2.5:1 (v/w). SOM was determined by wet digestion using the potassium dichromate method [38]. TN was determined using the micro-Kjeldahl method [39]. Inorganic N species (NH_4_−N and NO_3_−N) were extracted with 2 M KCl and detected on a continuous low analyzer (Skalar, Breda, Netherlands). TP was digested with HF−HClO_4_ and AP was extracted with sodium bicarbonate, respectively, which were determined using the molybdenum-blue method [40, 41]. TK was digested with HF−HClO_4_ and AK was extracted with ammonium acetate, respectively, which were detected by atomic absorption spectrophotometer [42]. Gravimetric SWC was measured by drying the soil for 48 h at 105 °C. The acid and alkaline phosphomonoesterase activities were assayed using *p*-nitrophenyl phosphate (*p*-NP) as the substrate with the buffer adjusted to pH 6.5 and 11.0, respectively [43]. After incubation, the absorption was measured at 405 nm, and acid and alkaline phosphomonoesterase activity were expressed as mg *p*-NP g^−1^ soil h^−1^.

### Lipid extraction and analysis

The biomasses of AMF and saprotrophic fungi were characterized by neutral lipid fatty acid (NLFA) and phospholipid fatty acid (PLFA) analysis, respectively [44]. Briefly, total lipids were extracted from 2 g freeze-dried soil samples with a mixture of chloroform, methanol, and citrate buffer (1:2:0.8, v/v/v), and then fractionated into neutral, glyco-, and phospho-lipids by silica acid columns. The neutral lipids and phospholipids were converted to methyl esters by alkaline methanolysis, and then quantified by a HP 6890 Series gas chromatograph (Hewlett–Packard, Wilmington, DE, USA). Identification was performed using fungal fatty acid standards and MIDI peak identification software (Microbial ID Inc., Newark, DE, USA). The methyl nonadecanoate (19:0) was used as internal quantitative standard. Fungal biomass was calculated by summing the abundance of specific biomarkers and expressed as nmol NLFA/PLFA g^–1^ dry soil. The biomarker NLFA 16:1ω5c was used as the indicator of AMF biomass, and the biomarkers PLFA 18:1ω9c and 18:2ω6,9c as saprotrophic fungal biomass [45, 46].

### Illumina sequencing and bioinformatic analysis

The soil DNA was extracted from 0.5 g samples using the DNeasy PowerSoil Kit (Qiagen, Hilden, Germany) following the manufacturer’s instructions. The extracted DNA was dissolved in tris-EDTA buffer and quantified by the ND-1000 spectrophotometer (NanoDrop Technologies, Wilmington, DE, USA). For the AMF and protistan communities, triplicate PCR amplifications of the 18S rRNA gene fragments were performed using the primer sets of AMV4.5NF/AMDGR [47] and TAReuk454FWD1/TAReukREV3 [48], respectively. The 8-bp barcode oligonucleotides were added to distinguish the amplicons from different soil samples. Reaction mixtures (20 μL) contained 2 μL of 10 × reaction buffer, 0.25 μL of each primer (10 μM), 2 μL of 2.5 mM dNTPs, 10 ng template DNA, and 0.4 μL FastPfu Polymerase. The PCR protocol was as follows: an initial pre-denaturation at 95 °C for 5 min, followed by 28 cycles of denaturation at 94 °C for 30 s, annealing at 60 °C for 30 s, and extension at 72 °C for 45 s; and a final extension at 72 °C for 10 min with a ramp of 3 °C s^−1^. All amplicons were cleaned and pooled in equimolar concentrations in a single tube, after which they were subjected to library preparation, cluster generation, and 300 bp paired-end sequencing on an Illumina MiSeq platform (Illumina, San Diego, CA, USA).

Raw sequences were quality screened and trimmed using the Quantitative Insights into Microbial Ecology (QIIME package version 1.9.1) pipeline [49]. Sequences that fully matched the barcodes were selected, and sequence processing was performed including quality trimming, demultiplexing, and taxonomic assignments. QIIME quality trimming was performed in accordance with the following criteria: (1) no ambiguous bases, and (2) the minimum sequence length of 283 bp (AMF) and 516 bp (protist) after trimming. The assembled reads were processed using de novo chimera detection in UCHIME [50]. Thereafter, the sequence reads from each sample were clustered to provide similarity-based operational taxonomic units (OTUs) that had 97% identity cutoffs [51]. Finally, the sequences were subjected to a similarity search using the MaarjAM AMF database and the Protist Ribosomal Reference database (PR2, v4.3), respectively [16, 52]. Prior to downstream analyses, AMF and protistan OTUs were extracted from the individual OTU table to represent the structure of soil AMF and protistan communities. For the 3% cutoff, 537 AMF and 2798 protistan OTUs were observed out of 313,584 and 480,107 high-quality sequences, respectively. Alpha diversity and Bray-Curtis distances for a principal coordinate analysis of AMF, fungivorous protist, and nematode communities were calculated after rarefying all samples to the same sequencing depth. Functional units of protists were categorized according to their feeding habits [26, 53].

### AMF colonization and root morphology

Plants from plots were randomly selected for the determination of AM root colonization (in percent) [54]. Briefly, roots of each plant were carefully washed with distilled water for three times in order to remove soil particles, and then cut into 1-cm-long fragments. Subsequently, root fragments were randomly selected and cleared in 10% KOH solution in a boiling water bath for 45 min. After rinsing with distilled water, root fragments were immersed in 1% HCl for 15 min, bleached in 10% hydrogen peroxide for 10 min. Then, roots were cleaned and stained for two hours in 0.02% (w/v) aniline blue solution at room temperature. Fifty root fragments per replicate were examined at × 100−400 magnification under a compound microscope for the presence of AM structures. AMF colonization was calculated as the percentage of the total root segments containing visible AMF structures.

Shoot biomass, root biomass, and grain yield of maize were measured immediately after harvest. We processed digital images of root system morphology using a desktop scanner and determined root length, surface area, average diameter, root volume, and number of tips, forks, and crossings using WinRhizo software (Regent Instruments, Québec, Canada). All measurements were expressed per g of root mass and scaled to a per m^2^ basis based on total standing root biomass (g m^−2^) at the plot level.

### Identification and isolation of nematode assemblages

Nematodes were extracted from 100 g fresh soil using the shallow dish method [55]. Four functional groups of nematode assemblages, including bacterivores, fungivores, plant parasites, and omnivores and predators, were identified based on known feeding habits, stoma, and esophageal morphology [9]. Nematode density was counted and expressed as nematode numbers per 100 g of dry weight soil.

Two kinds of fungivorous nematodes (*Aphelenchoides* and *Aphelenchus*) were separately picked out into 10 mM sterile phosphate buffer saline (pH 7.0) under a dissecting microscope according to morphological characteristics. These harvested nematodes were then introduced into 2% sodium hypochlorite solution for 30 s to avoid microbial interference from the body surface, then washed five times with sterile distilled water. AMF spores in the final wash water were isolated and enumerated by wet-sieving and sucrose gradient centrifugation [56, 57], and AMF abundance indicated by copy numbers of the 18S rRNA gene were quantified. Neither AMF spore nor AMF abundance was detected, suggesting that nematodes had been surface sterilized. In order to verify the predation of fungivorous nematodes on AMF, 30 individuals of *Aphelenchoides* or *Aphelenchus* were chosen and transferred into a 1.5 mL centrifuge tube under sterile conditions for DNA extraction.

### Quantitative polymerase chain reaction (qPCR) and reverse transcription-PCR (qRT-PCR)

Total DNA of surface-sterilized *Aphelenchoides* or *Aphelenchus* was extracted using a DNeasy Blood & Tissue Kit (Qiagen, Hilden, Germany) according to the manufacturer’s instructions. AMF abundance inside fungivorous nematodes was assessed by copy numbers of AMF-specific 18S rRNA gene using the same primers as described above. The qPCR assays were conducted in triplicate by using the fluorescent dye SYBR-Green approach on an ABI 7500 Sequence detection system (Applied Biosystems, Foster City, CA, USA). The standard curve for AMF was obtained using 10-fold serial dilutions (10^2^−10^8^ copies) of plasmid DNA carrying the corresponding gene fragment. Target DNA was successfully amplified from all samples with an efficiency of 95−107% and correlation coefficients higher than 0.99, except for negative controls. AMF abundance inside *Aphelenchoides* or *Aphelenchus* was calculated as the copy number of AMF 18S rRNA gene per nematode, respectively.

Root total RNA was isolated using RNA Plus (Takara, Dalian, China) with the guanidine thiocyanate extraction method. Then, 1.2% agarose gel and the NanoDrop ND-1000 spectrophotometer were used to determine quality and quantity RNA (NanoDrop Technologies, Wilmington, DE, USA), respectively. DNase was used to eliminate the potential trace of genomic DNA in RNA samples. Root RNA was reversely transcribed into cDNA as templates for RT-PCR using the Roche reverse transcription kit. The qRT-PCR was carried out on an ABI 7500 Sequence detection system (Applied Biosystems, Foster City, CA, USA). To support the notion that mycorrhizal colonization regulates mycorrhizal P acquisition in roots, the *ZmPht1;3* and *ZmPht1;6* genes encoding P transporter of the PHT1 family were monitored. The *ZmPHT1;3* and *ZmPHT1;6* genes were amplified with the primer pairs [58]. The expression level of the maize *Actin 1* gene was used as an internal control. The relative transcript level was normalized as percent of the corresponding actin transcript levels.

### Statistical analysis

One-way analysis of variance (ANOVA) was performed to assess the effects of manure treatments on soil properties, the AMF communities, the assemblages of fungivorous protists and nematodes, plant performance using Tukey’s HSD test in SPSS 23.0 software (SPSS, Chicago, IL, USA). All statistical analyses were conducted based on 12 samples (4 fertilization treatments × 3 replicates). Principal coordinate analysis (PCoA) was used to evaluate the Bray-Curtis distances of the AMF, protistan, and nematode community compositions under manure treatments [59]. We conducted the ‘capscale’ function of the R package vegan (version 3.1.2) to calculate the Bray-Curtis dissimilarities for PCoA and ‘permutest’ permutation-based testing for the calculation of the significance values [60].

To describe the complex co-occurrence patterns in mycorrhizal–fungivores networks, we constructed a correlation matrix by calculating multiple correlations and similarities with Co-occurrence Network (CoNet) inference [61]. The OTUs detected in more than three-fourths of the soil samples at the same depth were kept for the network construction. We transformed the distribution matrix of AMF, and fungivorous protists and nematodes into the relative abundance values. Then, we used an ensemble approach that combined four measurements, including Pearson and Spearman correlations and Bray-Curtis and Kullback-Leibler dissimilarities. A valid co-occurrence was considered a statistically robust correlation between species when the correlation coefficient (*r*) was > 0.8 or < − 0.8 and the *P* value was < 0.01. Those *P* values < 0.01 were adjusted by a testing correction using the Benjamini-Hochberg procedure to reduce the chances of obtaining false-positive results [62]. Co-occurrence networks were visualized via Gephi software [63]. Modules were defined as clusters of closely interconnected nodes (i.e., groups of co-occurring microbes) [64]. The microbial networks were searched to identify highly associated nodes (clique-like structures) using Molecular Complex Detection (MCODE) introduced for the Cytoscape platform [65]. We calculated the first principal component of the modules (module eigengene) in the standardized module expression data for the co-occurrence networks [66]. The correlations between soil properties, network module eigengenes, AMF colonization, the expression of *ZMPht1;6* gene, and plant performance were evaluated using Spearman’s rank correlation test.

Random forest tool was performed to quantitatively estimate the important predictors of AMF colonization and the expression of P transporter genes containing soil properties, AMF community, and the assemblages of fungivores. Random forest modeling was conducted using the randomForest package [67] and the model significance and predictor importance were determined using the A3R and rfPermute packages, respectively [68, 69]. Based on random forest analyses, the significant predictors were further chosen to perform a structural equation modelling (SEM) analysis. SEM analysis was applied to determine the direct and indirect contributions of soil properties and mycorrhizal-fungivore interactions to AMF colonization and plant productivity. SEM analysis was conducted via the robust maximum likelihood evaluation method using AMOS 20.0. A path indicated the partial correlation coefficient and interpreted the magnitude of the relationships between two parameters. Latent variables were used to integrate the effects of multiple conceptually related observed variables into a single-composite effect, aiding interpretation of model results. The SEM fitness was examined on the basis of a non-significant chi-square test (*P* > 0.05), the goodness-of-fit index, and the root mean square error of approximation [70].

## Supplementary information

**Additional file 1: Fig. S1.** Soil characteristics in the rhizosphere under manure treatments. **Fig. S2.** The alkaline and acid phosphomonoesterase activity in the rhizosphere under manure treatments. **Fig. S3.** The structure of arbuscular mycorrhizal fungi, fungivorous protist and nematode communities by principal coordinate analysis. **Fig. S4.** The ratios of arbuscular mycorrhizal fungi to plant biomass and fungivorous nematodes to plant biomass. **Fig. S5.** Correlation coefficients between arbuscular mycorrhizal fungi (AMF) community (biomass, diversity, and composition), fungivorous protists and nematodes, ALP activity, AMF colonization and the expression of *ZMPht1;6* gene. **Fig. S6.** Mean contribution of soil variables, arbuscular mycorrhizal fungi (AMF) community, protists, and nematodes to AMF colonization and expression of P transporter gene *ZMPht1;6* based on random forest modelling. **Table S1.** The characteristics of fine roots under four manure treatments.

## Data Availability

All databases of AMF and protistan communities generated in this study were available at GenBank’s Sequence Read Archive under Bioproject number PRJNA660819 and PRJNA623238, respectively.
