# Supplementary material for: Trophic interactions as determinants of the arbuscular mycorrhizal fungal community with cascading plant-promoting consequences
Source: Microbiome. 2020 Oct 2;8:142. doi: 10.1186/s40168-020-00918-6 (PMC7532650; doi:10.1186/s40168-020-00918-6)
Supplement: Supplementary file 2 — Additional file 1: Fig. S1. Soil characteristics in the rhizosphere under manure treatments. Fig. S2. The alkaline and acid phosphomonoesterase activity in the rhizosphere under manure treatments. Fig. S3. The structure of arbuscular mycorrhizal fungi, fungivorous protist and nematode communities by principal coordinate analysis. Fig. S4. The ratios of arbuscular mycorrhizal fungi to plant biomass and fungivorous nematodes to plant biomass. Fig. S5. Correlation coefficients between arbuscular mycorrhizal fungi (AMF) community (biomass, diversity, and composition), fungivorous protists and nematodes, ALP activity, AMF colonization and the expression of ZMPht1;6 gene. Fig. S6. Mean contribution of soil variables, arbuscular mycorrhizal fungi (AMF) community, protists, and nematodes to AMF colonization and expression of P transporter gene ZMPht1;6 based on random forest modelling. Table S1. The characteristics of fine roots under four manure treatments. [file 40168_2020_918_MOESM1_ESM.docx]

**SUPPLEMENTARY INFORMATION FOR**

**Trophic interactions as determinants of the arbuscular mycorrhizal fungal community with cascading plant-promoting consequences**

Yuji Jiang^1,^*, Lu Luan^1^, Kaijie Hu^2^, Manqiang Liu^3^, Ziyun Chen^1^, Stefan Geisen^4,^*, Xiaoyun Chen^3^, Huixin Li^3^, Qinsong Xu^2^, Michael Bonkowski^5^, Bo Sun^1,^*

**Corresponding authors:**

Yuji Jiang yjjiang@issas.ac.cn

Stefan Geisen stefan.geisen@wur.nl

Bo Sun bsun@issas.ac.cn

**Supplementary Figures S1–S6**

**Fig. S1** Soil characteristics in the rhizosphere under manure treatments.

**Fig. S2** The alkaline and acid phosphomonoesterase activity in the rhizosphere under manure treatments.

**Fig. S3** The structure of arbuscular mycorrhizal fungi, fungivorous protist and nematode communities by principal coordinate analysis

**Fig. S4** The ratios of arbuscular mycorrhizal fungi to plant biomass and fungivorous nematodes to plant biomass.

**Fig. S5** Correlation coefficients between arbuscular mycorrhizal fungi (AMF) community (biomass, diversity, and composition), fungivorous protists and nematodes, ALP activity, AMF colonization and the expression of *ZMPht1;6* gene

**Fig. S6** Mean contribution of soil variables, arbuscular mycorrhizal fungi (AMF) community, protists, and nematodes to AMF colonization and expression of P transporter gene *ZMPht1;6* based on random forest modelling.

**Supplementary Table S1**

**Table S1** The characteristics of fine roots under four manure treatments.

**Supplementary Figures**


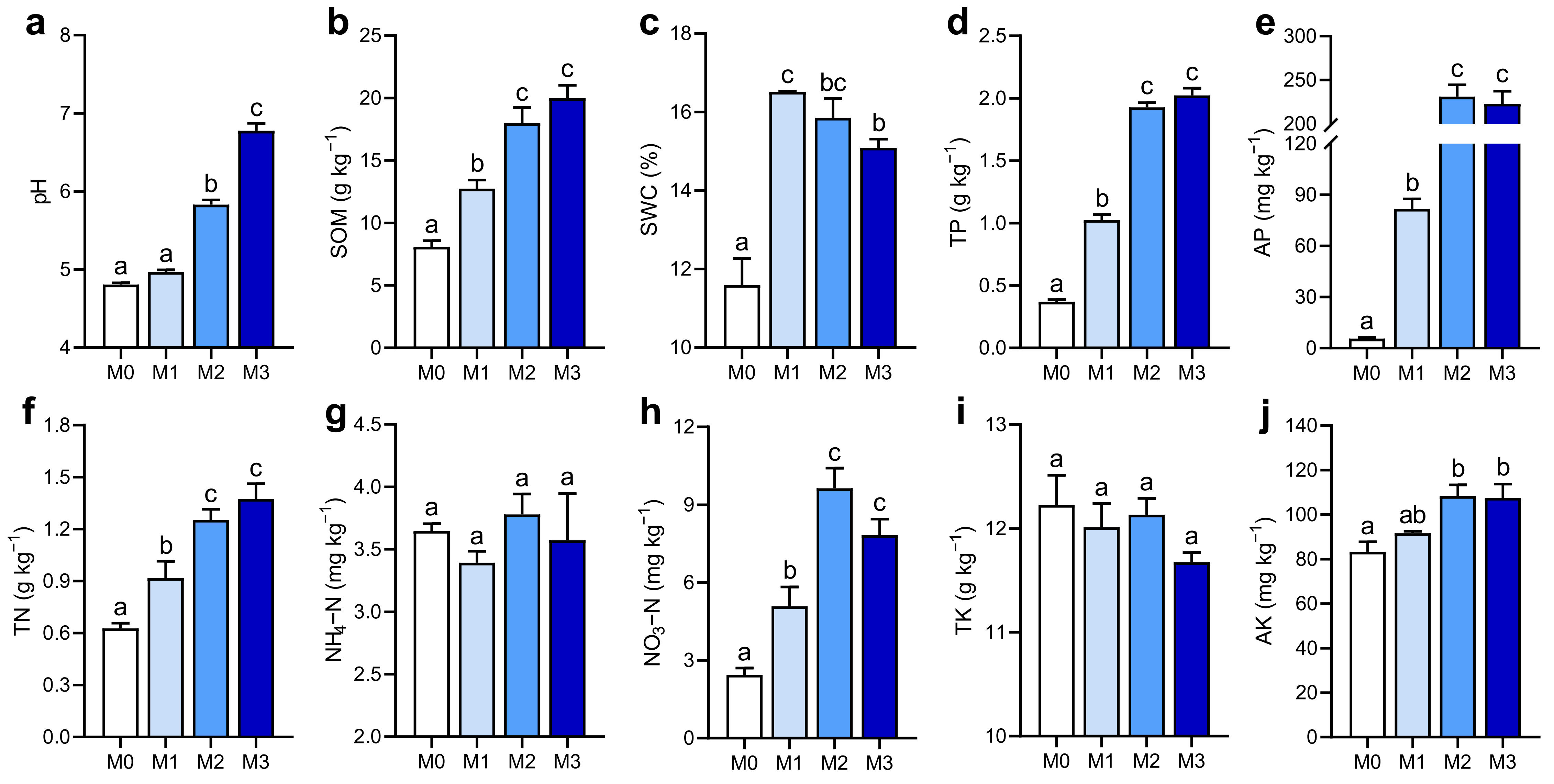


**Fig. S1** Soil characteristics in the rhizosphere under manure treatments, including pH (**a**), soil organic matter (SOM, **b**), soil water content (SWC, **c**), total phosphorus (TP, **d**), available phosphorus (AP, **e**), total nitrogen (TN, **f**), ammonium nitrogen (NH_4_−N, **g**), nitrate nitrogen (NO_3_−N, **h**), total potassium (TK, **i**), available potassium (AK, **j**). Bars (n=3) with different lowercase letters indicate significant differences as revealed by Tukey’s HSD tests (*P* < 0.05). M0, no manure; M1, low manure; M2, high manure; M3, high manure plus lime.


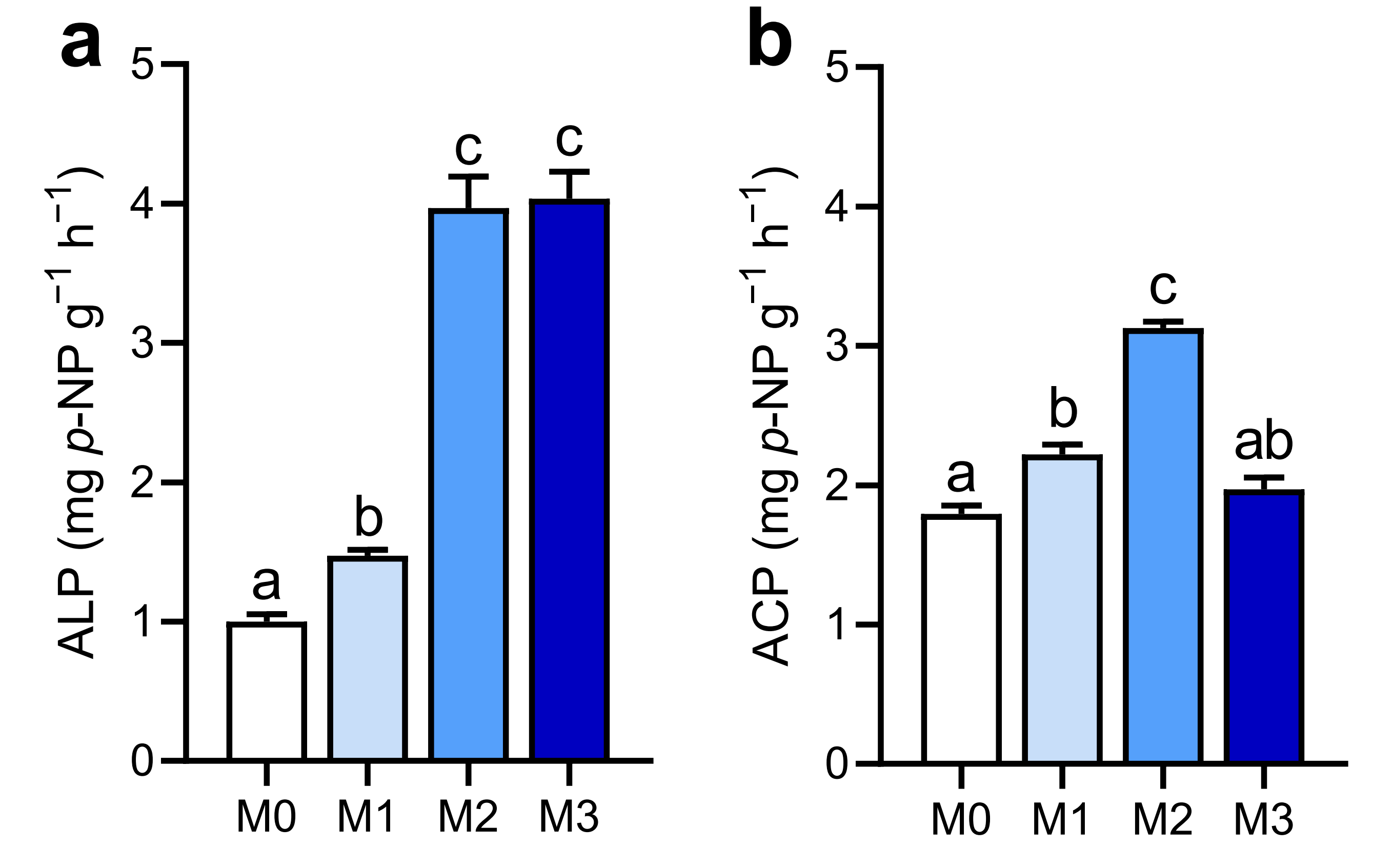


**Fig. S2** The alkaline (ALP, **a**) and acid (ACP, **b**) phosphomonoesterase activity in the rhizosphere under manure treatments. Error bars represent standard errors of three replicates. Bars (n=3) with different lowercase letters indicate significant differences as revealed by Tukey’s HSD tests (*P* < 0.05). M0, no manure; M1, low manure; M2, high manure; M3, high manure plus lime.


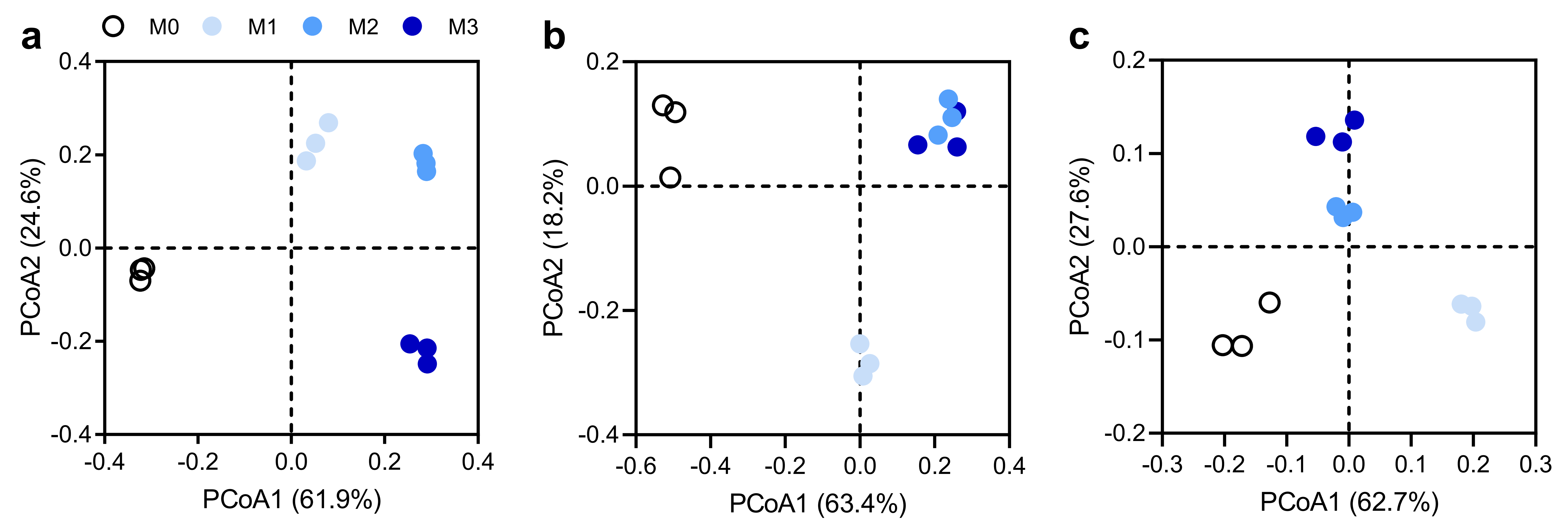


**Fig. S3** The structure of arbuscular mycorrhizal fungi (AMF, **a**), fungivorous protist (**b**) and nematode (**c**) communities by principal coordinate analysis (PCoA), which is constrained by manure treatments based on Bray-Curtis distances. M0, no manure; M1, low manure; M2, high manure; M3, high manure plus lime.


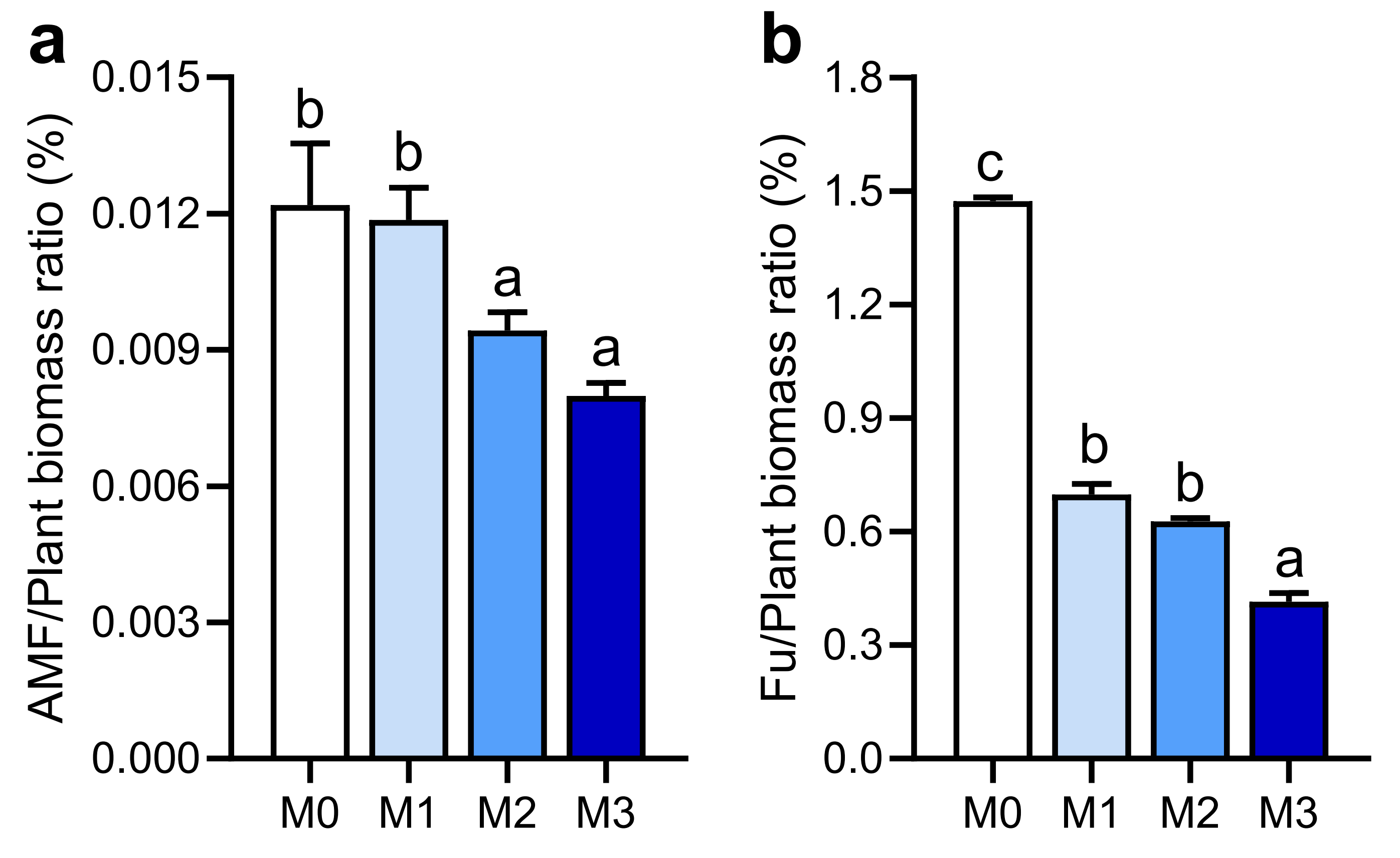


**Fig. S4** The ratios of arbuscular mycorrhizal fungal (AMF) biomass (nmol NLFA g^−1^ soil) to plant biomass (**a**) and the density of fungivorous nematodes (Fu, ind 100 g^−1^ dry soil) to plant biomass (**b**). Bars (n=3) with different lowercase letters indicate significant differences as revealed by Tukey’s HSD tests (*P* < 0.05). Plant biomass is the sum of root, shoot and grain biomasses. M0, no manure; M1, low manure; M2, high manure; M3, high manure plus lime.


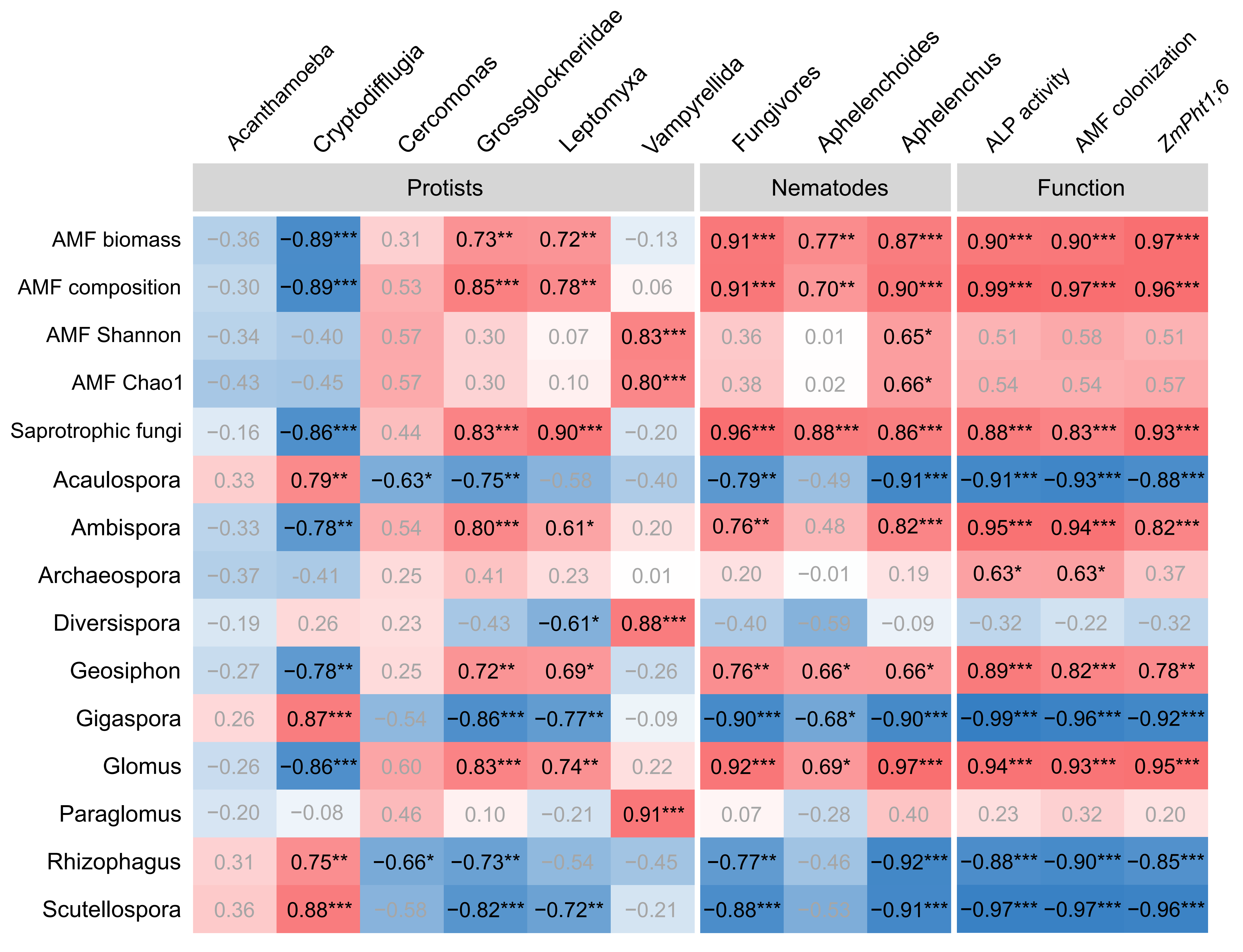


**Fig. S5** Correlation coefficients between arbuscular mycorrhizal fungi (AMF) community (biomass, diversity, and composition), saprotrophic fungi (biomass), fungivorous protists and nematodes, ALP activity, AMF colonization and the expression of *ZMPht1;6* gene. Relative abundances of AMF, and fungivorous protist and nematode communities were used for calculating these correlations. Correlation coefficients were calculated based on 12 samples (4 fertilization treatments × 3 replicates). Black values denote the significant relationships. *** *P* < 0.001; ** *P* < 0.01; * *P* < 0.05.


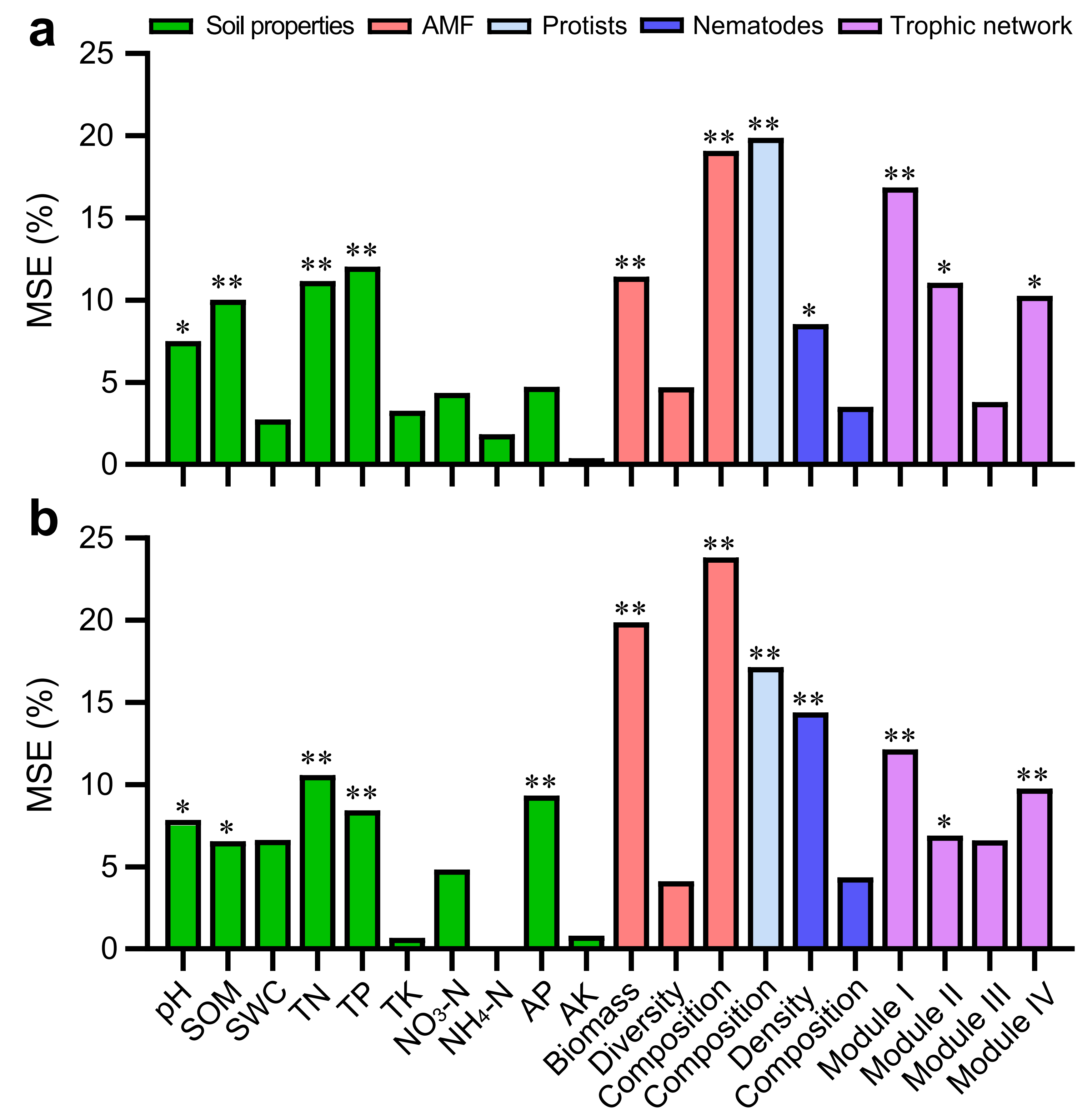


**Fig. S6** Mean contribution (% of increased mean square error, MSE) of soil variables, arbuscular mycorrhizal fungi (AMF) community, protists, and nematodes to AMF colonization (**a**) and expression of P transporter gene *ZMPht1;6* (**b**) based on random forest modelling. Random forest modelling was performed based on 12 samples (4 fertilization treatments × 3 replicates). Soil properties include pH, soil organic matter (SOM), soil water content (SWC), total nitrogen (TN), total phosphorus (TP), total potassium (TK), ammonia nitrogen (NH_4_−N), nitrate nitrogen (NO_3_−N), available phosphorus (TP), and available potassium (TK). The AMF community includes biomass, diversity (Shannon index), and composition (first principal coordinates, PCoA1). Protist assemblage includes composition (PCoA1). Nematode assemblage includes density and composition (PCoA1). Four modules in the trophic co-occurrence network between AMF, protist and nematodes are represented by module eigengenes.

**Supplementary Table**

**Table S1** The characteristics of fine roots under four manure treatments^a^.

| Fine root metric | M0 | M1 | M2 | M3 |
| --- | --- | --- | --- | --- |
| Root length (cm) | 196.09±11.33a | 318.14±4.31b | 417.96±20.17c | 446.28±14.71 |
| Projected area (cm^2^) | 17.53±0.12a | 37.52±2.11b | 41.36±0.99b | 51.99±1.39c |
| Surface area (cm^2^) | 55.07±0.38a | 117.87±4.65b | 129.94±3.12b | 163.32±3.53c |
| Average diameter (mm) | 0.84±0.01a | 0.92±0.01a | 1.32±0.01c | 1.19±0.02b |
| Root volume (cm^3^) | 1.23±0.01a | 2.65±0.07b | 4.22±0.15c | 4.75±0.19c |
| Tips | 105±7a | 389±16b | 450±22c | 475±25c |
| Forks | 336±13a | 821±33b | 1020±46c | 1089±22c |
| Crossings | 19±1a | 57±5b | 82±12c | 80±7c |

**a.** Mean values of all dates are accompanied by standard error (n=3). Values in the same row followed by a lowercase letter indicate significant differences according to Tukey’s HSD tests (*P* < 0.05). M0, no manure; M1, low manure; M2, high manure; M3, high manure plus lime.
